# Targeting PAR2 Overcomes Gefitinib Resistance in Non-Small-Cell Lung Cancer Cells Through Inhibition of EGFR Transactivation

**DOI:** 10.3389/fphar.2021.625289

**Published:** 2021-04-22

**Authors:** Yuhong Jiang, Xin Zhuo, Xiujuan Fu, Yue Wu, Canquan Mao

**Affiliations:** School of Life Science and Engineering, Southwest Jiaotong University, Chengdu, China

**Keywords:** protease-activated receptor 2, epidermal growth factor receptor-tyrosine kinase inhibitors, drug resistance, non-small-cell lung cancer cells, transactivation

## Abstract

Drug resistance can notably restrict clinical applications of gefitinib that is a commonly used EGFR-tyrosine kinase inhibitors (EGFR-TKIs) for non-small cell lung cancer (NSCLC). The attempts in exploring novel drug targets and reversal strategies are still needed, since gefitinib resistance has not been fully addressed. Protease-activated receptor 2 (PAR2), a G protein-coupled receptor, possesses a transactivation with EGFR to initiate a variety of intracellular signal transductions, but there is a lack of investigations on the role of PAR2 in gefitinib resistance. This study established that protease-activated receptor 2 (PAR2), actively participated in NSCLC resistant to gefitinib. PAR2 expression was significantly up-regulated when NSCLC cells or tumor tissues became gefitinib resistance. PAR2 inhibition notably enhanced gefitinib to modulate EGFR transactivation, cell viability, migration and apoptosis in gefitinib-sensitive and-resistant NSCLC cells, suggesting its reversal effects in gefitinib resistance. Meanwhile, the combination of a PAR2 inhibitor (P2pal-18S) and gefitinib largely blocked ERK phosphorylation and epithelial-mesenchymal transition (EMT) compared to gefitinib alone. Importantly, we probed its underlying mechanism and uncovered that PAR2 blockade sensitized gefitinib and reversed its resistance mainly via β-arrestin-EGFR-ERK signaling axis. These effects of PAR2 inhibition were further confirmed by the *in vivo* study which showed that P2pal-18S reactivated gefitinib to inhibit tumor growth via restricting ERK activation. Taken together, this study could not only reveal a new mechanism of receptor-mediated transactivation to modulate drug resistance, but also provide a novel drug target and direction for overcoming gefitinib resistance in NSCLC.

## Introduction

Non-small cell lung cancer (NSCLC) accounts for approximately 85% of lung cancers which is the leading cause of cancer-related death worldwide (Gridelli et al., 2015; Herbst et al., 2018). Since an epidermal growth factor receptor (EGFR) activating mutation has been identified as one of the most common gene mutations in lung cancer, EGFR-tyrosine kinase inhibitors (EGFR-TKIs) became first-line molecular targeted therapies for EGFR-mutated NSCLC patients (Network, 2014). Gefitinib as the first-generation EGFR-TKI has markedly prolonged the overall survival of NSCLC patients; however, drug resistance has been eventually harboured leading to the ultimate failure of clinical treatments (Hirsch et al., 2016). There are a variety of resistance mechanisms, including second-site mutations (e.g., EGFR-T790M gatekeeper mutation) (Yun et al., 2008), compensatory downstream signaling activation (e.g., MEK/ERK, PI3K/AKT) (Shou et al., 2016; Rotow and Bivona, 2017), parallel bypass signaling activation (other receptor tyrosine kinases, RTKs, e.g., MET, FGFR, IGFR) (Rotow and Bivona, 2017; Raoof et al., 2019) and histological transformation (e.g., epithelial-to-mesenchymal transition, EMT) (Marcucci et al., 2016; Weng et al., 2019). Although the third-generation EGFR-TKI, osimertinib, has been successfully developed to overcome EGFR-T790M mutated resistance to gefitinib (Jänne et al., 2015), other resistance mechanisms have not been clinically well-addressed due to its diverse and complicated characteristics. Thus, it is an urgent need for exploring novel drug strategies targeting NSCLC resistant to gefitinib depending on other receptors, signaling pathways or histological changes.

G protein-coupled receptors (GPCRs) as the most widely studied drug targets have been reported to exhibit a transactivation with EGFR, i.e., GPCR ligands can activate EGFR to stimulate downstream signaling 2 decades ago (Daub et al., 1996). Protease-activated receptor 2 (PAR2) as a GPCR itself can facilitate Ca^2+^, MEK/ERK, AKT, PI3K and mTOR signaling pathways to promote a variety of tumor cell functions (Huang et al., 2013; Jiang et al., 2018; Tsai et al., 2018; Pawar et al., 2019). Meanwhile, PAR2 can transactivate EGFR and subsequently trigger MEK/ERK or AKT signaling, cooperatively modulating cell migration, apoptosis or proliferation in colon (Darmoul et al., 2004), pancreatic (Shi et al., 2013), gastric (Caruso et al., 2006), lung (Michel et al., 2014) and ovarian cancers (Jiang et al., 2020). PAR2 blockade can significantly impair cell migration and EMT via ERK signaling in lung cancer (Tsai et al., 2018) whereas the dysregulation of ERK signaling and EMT are considered as molecular mechanisms of gefitinib resistance in NSCLC (Tricker et al., 2015; Becker et al., 2019; Weng et al., 2019), suggesting the potential involvement of PAR2-mediated signaling in lung tumor progression and drug resistance. Notably, mutational activations of other RTKs have also been identified to initiate gefitinib resistance since these receptors can exhibit parallel signaling pathways required for NSCLC cell survival (Rotow and Bivona, 2017). Similarly, PAR2-mediated EGFR transactivation can regulate EGFR-related signaling and therefore we hypothesized that this transactivation might also bypass gefitinib-induced EGFR blockade to modulate NSCLC resistant to gefitinib. A study on expression analysis of 384 GPCRs in gefitinib resistance has also showed that the level of PAR2 expression was largely higher in gefitinib-resistant NSCLC cells than sensitive cells (Kuzumaki et al., 2012), further indicating that PAR2 might be positively related to the development of gefitinib resistance. However, classical strategies mainly focus on modulation of resistance-related EGFR mutations, RTKs and compensatory signaling pathways, and few literatures reported about the role of other receptor families, including PAR2, in gefitinib resistance upon EGFR transactivation.

In this study, we aimed to investigate whether inhibition of PAR2 could overcome gefitinib resistance in NSCLCs as well as its underlying mechanism *in vitro* and *in vivo*. Here, we first showed that combination of a PAR2 antagonist, P2pal-18S, and gefitinib can cooperatively impair phosphorylation of EGFR to confirm the transactivation between PAR2 and EGFR. Furthermore, inhibition of PAR2 augmented the effect of gefitinib in cell proliferation, migration and apoptosis in gefitinib-sensitive and -resistant NSCLC cells while targeting PAR2 promoted gefitinib to modulate ERK signaling and EMT. PAR2 blockade potentiated gefitinib in attenuating NSCLC cell functions via β-arrestin-ERK signalling axi upon EGFR transactivation. Alternatively, *in vivo* xenograft study of combination of P2pal-18S and gefitinib confirmed the effect and mechanism of targeting PAR2 in gefitinib resistance. Taken together, our study revealed the promising role of PAR2 in NSCLC cell resistant to gefitinib and targeting PAR2 maybe as a potential therapeutic strategy for reversing gefitinib resistance and improving EGFR-TKI therapy in NSCLC patients.

## Materials and Methods

### Materials

Gefitinib (99%) was obtained from Aladdin (Shanghai, China) and P2pal-18S (palmitate-RSSAMDENSEKKRKSAIK-NH_2_, 98%) was custom-made by Apeptides (Shanghai, China). Mitomycin C (99%) and FR180204 (100%) were purchased from Selleckchem (Houston, TX, United States) while Barbadin (99%) were obtained from MedChemExpress (Monmouth Junction, NJ, United States). The primary antibodies used against PAR2, total or phospho-EGFR, total or phospho-ERK as well as the secondary antibodies, HRP-conjugated anti-rabbit IgG, were purchased from Cell Signaling Technology (Boston, MA, United States). The primary antibodies used against Bcl-2, Bax, E-cadherin and vimentin were obtained from Abcam (Cambridge, MA, United States).

### Cell Cultures and Construction of Gefitinib-Resistant Cells

Human non-small lung cancer cell line PC-9 (KeyGEN BioTech, Nanjing, China) and A549 (the Cell Bank of the Chinese Academy of Science, Shanghai, China) were cultured at 37°C in DMEM or RPMI-1640 medium (Invitrogen, Carlsbad, CA, United States) containing 10% FBS and 1% penicillin/streptomycin in a humidified 5% CO_2_ incubator. To establish the gefitinib-resistant cells, PC-9 cells was exposed to gradually increasing concentrations of gefitinib as described previously (Shou et al., 2016). The gefitinib-resistant PC-9 cells (PC-9-GR) can proliferate normally in the presence of 5 mM gefitinib. The resistance index (RI) was calculated using formula: RI = IC_50_(PC-9-GR cells)/IC_50_(PC-9 cells).

### Cell Viability CCK8 Assays

Cells (5×10^4^ cells/mL) were seeded overnight and were then treated with compounds for 48 h. After that, Cells were incubated with 10 μL CCK-8 solution (KeyGEN BioTECH, Nanjing, China) for another 4 h. The absorbance was measured at 450 nm by using a microplate reader (Synergy H1, Biotek, United States).

### *In vitro* Scratch Assay

Cells (3 × 10^5^ cells/well) were seeded at 24 well-plate overnight and a scratch was created in the center of the well using a P200 micropipette tip. Cells were washed with PBS three times to remove floating cells and incubated with compounds. Scratch size were photographed at 0 and 48 h, using an inverted microscope (IX71, Olympus, Japan). The scratch gap size was measured using ImageJ software and was defined as the scratch size at 48 h divided by the initial gap area at 0 h.

### Transwell Migration Assay

Cell migration was measured by a transwell system (polycarbonate filter insert with 8 µm pore size membrane, Corning Inc., NY, United States). Cells were seeded at 5 × 10^5^/insert in the upper chamber of transwell overnight. Compounds were added to the upper chamber while medium with 2% FBS were added to the bottom chamber. After 24 h, cells on the top membrane were removed gently using cotton swabs and washed with PBS. Cells on the underside of the membrane were fixed in 4% paraformaldehyde and was then stained with 0.1% crystal violet. Migrated cells was photographed by an inverted brightfield microscope (BX53, Olympus, Japan) and was counted using ImageJ software.

### AO/EB Fluorescence Staining

Cells (5 × 10^5^ cells/well) were seeded at six well-plate and treated with P2pal-18S or gefitinib in serum-free medium for 48 h at 37°C. After that, the mixed AO/EB solutions (Solarbio, Bejing, China) were added to cells and incubated for 5 min in dark. Cell morphology was examined under a fluorescence microscope (IX71, Olympus).

### mRNA Isolation and Real Time-PCR

RNA was isolated using Trizol reagent (Invitrogen). Total RNA (1 µg) was reverse-transcribed by Revert PrimeScript RT Enzyme Kit (TaKaRa, Shiga, Japan). RT-PCR was then performed by a Lightcycler96 instrument (Roche Diagnostics, Switzerland). Genes were amplified for 40 cycles. The cycling conditions were 94°C for 10 min, followed by 40 cycles of 94°C for 30 s, 60°C for 40 s and 72°C for 20 s. Relative-gene expression was normalized against *Actin*. All primer sequences are described in Supplementary Table S1.

### Western Blot

Cells (5 × 10^5^ cells/well) were seeded overnight and then stimulated with compounds after serum-starve. Whole-cell lysates were prepared using RIPA lysis buffer (Beyotime, Shanghai, China) supplemented with 1% PMSF (Sigma). Each sample with equal loading was separated by 6–12% PAGE gel and then transferred to a nitrocellulose filter membrane. After blocked with 5% skim milk for 2 h, membranes were incubated with the primary antibodies (1:1000) and secondary antibodies conjugated to HRP (1:3000). Exposure times were varied to eliminate signal saturation while GAPDH, total EGFR or ERK was loading control. Densitometric analysis of bands was calculated with ImageJ software.

### Animal Experiments

All animals were kept in a specific pathogen-free facility and treated with humane care with approval from the Animal Use and Care Committee of Southwest Jiaotong University. For the xenograft studies, PC-9-GR cells (5 × 10^6^) were injected subcutaneously into the back of the right forelimbs of five-week-old male Balb/c nude mice (Chengdu Dashuo Laboratory Animal Technology, China). Once the tumor size reached ∼200 mm^3^, mice were randomized to four groups treated with vehicle, P2pal-18S (i.m., every two days), gefitinib (p.o., daily), or P2pal-18S and gefitinib. The tumor volume of mice was monitored twice a week and calculated using the formula V= (length × width^2^)/2. At indicated time points, mice were sacrificed, and tumor tissues were isolated. After fixed with 4% paraformaldehyde and embedded in paraffin, tumor tissues were detected by H&E staining for histopathological examination while levels of PAR2, total or phospho-ERK in tumor were detected by immunohistochemistry assay and observed under a light microscope (BX53, Olympus, Japan). mRNA and protein expressions of PAR2, total or phospho-ERK, E-cadherin or vimentin were also analyzed by RT-PCR and Western blot, respectively.

### Statistical Analysis

Data were plotted and analyzed using GraphPad Prism 8.0 (GraphPad Software, San Diego, CA, United States). Data point represents mean ± SEM (*n* ≥ 3) unless otherwise stated. Statistical significance of intergroup differences was calculated using one-way ANOVA analysis (*p < 0.05, **p < 0.01, ***p < 0.001).

## Results

### Protease-Activated Receptor 2 Inhibition Sensitized Non-Small Cell Lung Cancer Cells to Gefitinib Upon Epidermal Growth Factor Receptor Transactivation

**FIGURE 1:**
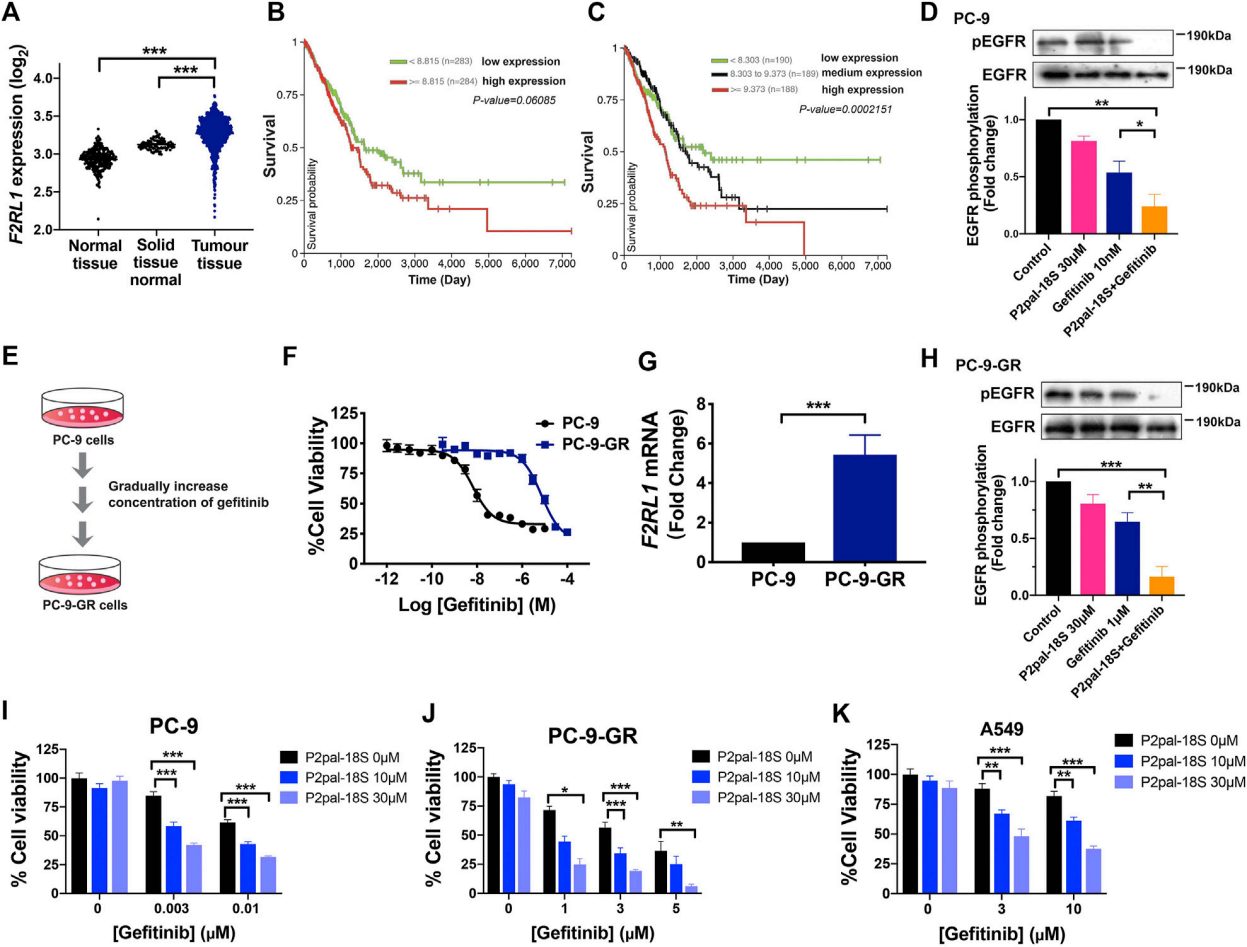
PAR2 was associated with tumor progression while PAR2 inhibition overcame gefitinib resistance in NSCLCs. **(A)** PAR2 (F2RL1) expression in lung tumor tissues from TCGA database (*n* = 1,011) is higher than normal lung tissues from GTE_X_ database (*n* = 288) or solid tissue normal samples from TCGA (*n* = 109); **(B–C)**. The Kaplan-Meier survival analysis of PAR2 expressions and TCGA lung adenocarcinoma (LUAD, *n* = 576) using UCSC Xena online tools; **(D)**. The synergic effect of a PAR2 inhibitor (P2pal-18S, 30 μM) and gefitinib (10 nM) in attenuating phosphorylation of EGFR in PC-9 cells; **(E–F)**. The gefitinib-resistant cells (PC-9-GR) were constructed and showed much less sensitive to gefitinib than PC-9 cells; **(G)**. PAR2 (F2RL1) expression was increased in PC-9-GR cells; **(H)**. P2pal-18S promoted gefitinib to inhibit phosphorylation of EGFR in PC-9-GR, detected by western blot. The densitometric analysis of each band was calculated with ImageJ software; **(I–K)**. Different concentrations of P2pal-18S augmented the growth inhibitory effect of gefitinib in PC-9 **(I)**, PC-9-GR **(J)**, A549 cells **(K)**. *p < 0.05, **p < 0.01, ***p < 0.001.

To probe the role of PAR2 in human NSCLC, we first compared the expression level of PAR2 (F2RL1) in human lung tumor tissues and normal lung tissues from The Cancer Genome Atlas (TCGA) and The Genotype-Tissue Expression (GTE_X_) databases using online analysis tools (Goldman et al., 2018). PAR2 expression was notably higher in human lung tumor tissues than that in normal lung tissues samples derived from peri-tumour tissues or lung tissues of persons without cancer (Figure 1A). Similarly, in the Gene Expression Omnibus (GEO) databases, lung tumor tissues exhibited up-regulation of PAR2 expression compared to normal tissues (Supplementary Figure S1). As shown in Figures 1B,C, there was a highly positive correlation between PAR2 expression and tumor progression of lung adenocarcinoma patients in Kaplan-Meier survival plots, revealing its active participations in lung cancer. It has also been discovered that PAR2 could not only itself mediate tumor progression but also transactive EGFR to cooperatively modulate cancer cell functions (Michel et al., 2014; Pawar et al., 2019). Consistent with previous findings, a PAR2 inhibitor, P2pal-18S, and gefitinib cooperatively attenuating EGFR activation in PC-9 cells (Figure 1D), indicating a transactivation between PAR2 and EGFR in NSCLC cells. We hypothesized that targeting PAR2 could reverse NSCLC cells resistance to gefitinib upon the receptor transactivation.

To test this hypothesis, we constructed a gefitinib-resistant NSCLC cells, PC-9-GR, by gradually increasing concentration of gefitinib in PC-9 cells (Figures 1E,F). PC-9-GR cells (IC_50_ 9.5 μM, pIC_50_ 5.2 ± 0.1) possessed more resistance (RI = 872) to gefitinib than PC-9 cells (IC_50_ 10.9 nM, pIC_50_ 7.8 ± 0.1), while A549 cells showed its primary resistance to gefitinib (IC_50_ 2.1 μM, pIC_50_ 5.0 ± 0.1) (Supplementary Figure S2A). Similar to our prior patient data analysis, the expression level of PAR2 was significantly elevated in PC-9-GR cells with increasing gefitinib resistance (Figure 1G). Due to drug resistance, gefitinib almost lost its capacity of blocking EGFR activation in PC-9-GR whereas addition of P2pal-18S largely promoted gefitinib to inhibit phosphorylation of EGFR (∼3-fold increase, Figure 1H). To further test whether PAR2 sensitize NCSLC cells to gefitinib, PC-9, PC-9-GR and A549 cells were treated with gefitinib at different concentrations in the presence of P2pal-18S at 10 μM or 30 μM. The incubation time of compound incubation was also studied and 48 h was selected as the optimal time point (Supplementary Figures S2B,C). As shown in Figures 1I–K, P2pal-18S significantly augmented gefitinib inhibition (∼70–90%) in sensitive cells PC-9, acquired resistant cells PC-9-GR and primary resistant cells A549 in dose-dependent manner, revealing the synergic effect between PAR2 and EGFR inhibitors. Importantly, no matter what concentrations of inhibitors were in Figures 1I,J, the inhibitory effect by the combination of P2pal-18S and gefitinib was ∼2-fold more than gefitinib alone in PC-9, while addition of P2pal-18S into gefitinib treatment triggered nearly a 3-fold increase in attenuating cell viability of PC-9-GR. It indicated that the sensitization effect of PAR2 blockade was more potent in PC-9-GR cells than in PC-9 cells. Therefore, with overexpression of PAR2 in NSCLC cells, PAR2 blockade sensitized gefitinib to attenuate EGFR activation and lung cancer cell viability.

### Protease-Activated Receptor 2 Inhibition Potentiated Gefitinib to Modulate Non-Small Cell Lung Cancer Cell Migration and Apoptosis

**FIGURE 2:**
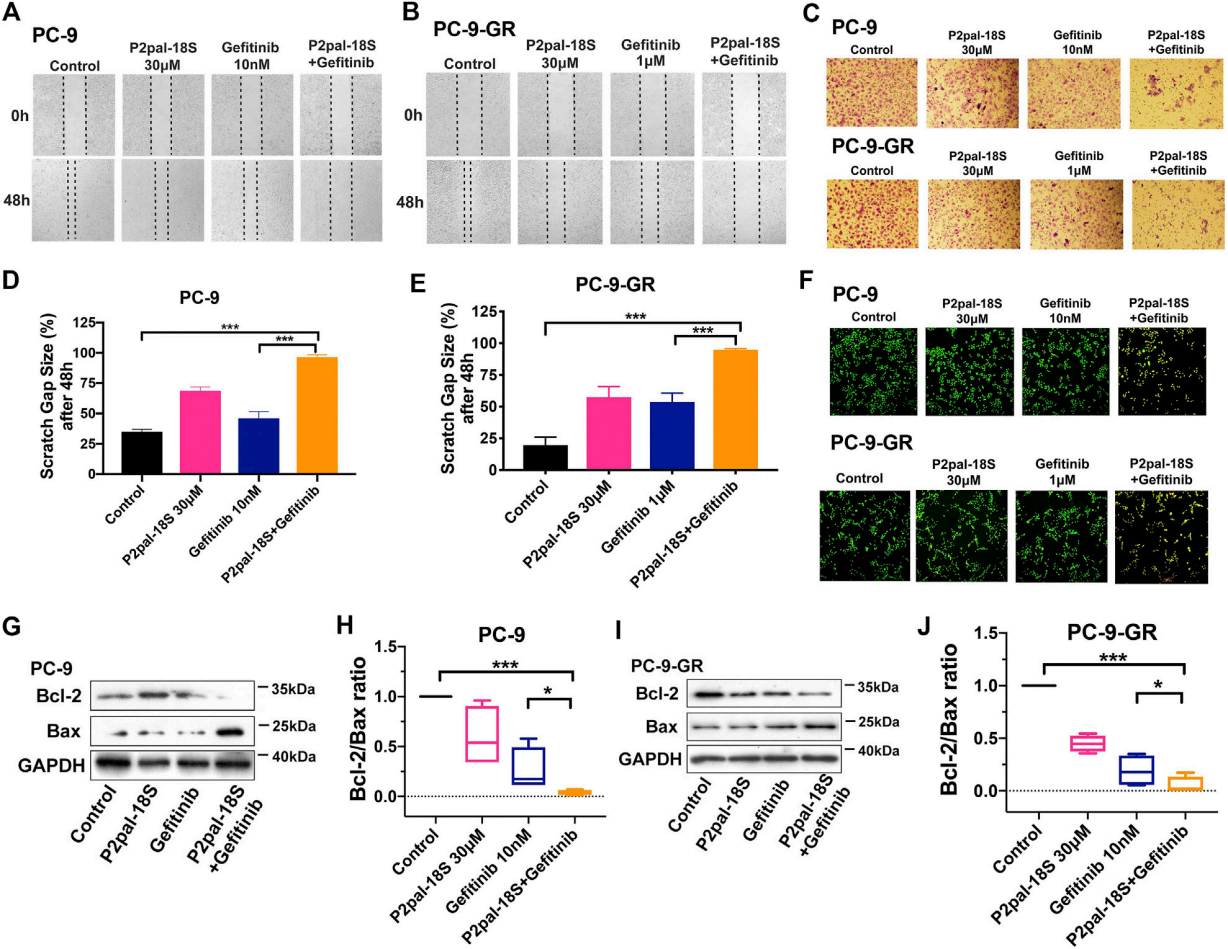
PAR2 augmented gefitinib-mediated NSCLC cell migration and apoptosis in both PC-9 and PC-9-GR cells. **(A-B)**. P2pal-18S and gefitinib showed a synergic effect for attenuating migration in *in-vitro* scratch assay; **(C)**. P2pal-18S enhanced gefitinib to inhibit cell migration detected by a transwell platform; **(D–E)**. The quantitative analysis of scratch gap sizes was calculated for different treatments; **(F)**. P2pal-18S facilitated gefitinib-induced apoptosis by AO/EB staining; **(G–J)**. With treatment of P2pal-18S and gefitinib, protein expressions of Bcl-2 and Bax were detected by western blot **(G,I)** and the ratio of Bcl-2 and Bax was calculated **(H,J)**. *p < 0.05, ***p < 0.001.

Apart from cell ability, we tested the reversal effect of PAR2 inhibition in cell migration and apoptosis. We found that there was a strong synergic effect of P2pal-18S and gefitinib in PC-9 and PC-9-GR cells by *in-vitro* scratch wound healing and transwell migration assay (Figures 2A–E), indicating that PAR2 inhibition exerted a significant enhancement of gefitinib in inhibiting NSCLC cell migration. Furthermore, as shown in Figure 2F, the increasing numbers of yellow-green or orange stained apoptotic/dead cells were observed in cells treated with combination of P2pal-18S and gefitinib whereas there were a large number of live cells (green) in the gefitinib treatment determined by the AO/EB fluorescence staining (Liu et al., 2015; Qi et al., 2020; Zhang et al., 2018). This effect of PAR2 blockade in NSCLC cell apoptosis was further confirmed by detection of Bcl-2 and Bax expressions (Figures 2G–J). Bcl-2/Bax ratio has been considered as a marker for cell apoptosis, since the decrease of Bcl-2 expression and increase of Bax expression represent cancer cells under apoptosis process. P2pal-18S largely promoted gefitinib to up-regulate Bax expression and down-regulate Bcl-2 expression in PC-9 and PC-9-GR cells, and therefore the Bcl-2/Bax ratio was notably reduced in NSCLC cells treated with P2pal-18S and gefitinib. Additionally, Annexin-FITC/PI staining results further confirmed that P2pal-18S could significantly enhance gefitinib to stimulate early or late apoptosis of PC-9 and PC-9-GR cells (Supplementary Figure S3). Thus, it indicated that PAR2 inhibition also facilitated gefitinib to limit NSCLC cell functions vs modulating cell migration and apoptosis.

### Protease-Activated Receptor 2 Inhibition Reversed EMT to Sensitize Non-Small Cell Lung Cancer Cells to Gefitinib

**FIGURE 3:**
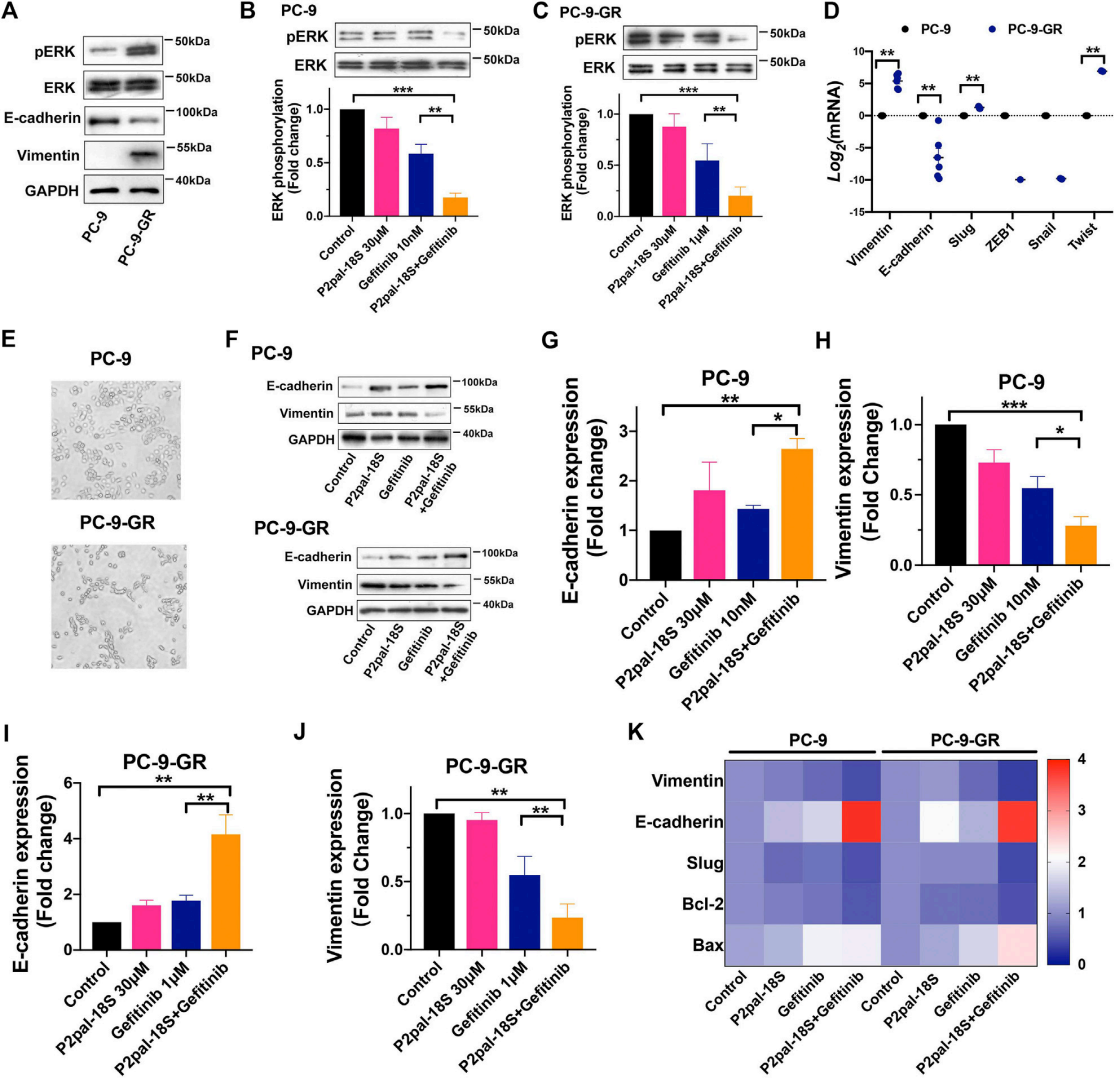
Targeting PAR2 enhanced gefitinib inhibition in ERK phosphorylation and EMT. **(A)**. Protein expressions of total or phosph-ERK, E-cadherin and vimentin were increased in PC-9-GR cells; **(B–C)**. P2pal-18S promoted gefitinib to inhibit phosphorylation of ERK in PC-9 **(B)** and PC-9-GR cells **(C)**; **(D)**. EMT-related gene expressions were detected and compared in gefitinib-sensitive and -resistant cells; **(E)**. The morphology of PC-9 and PC-9-GR cells was observed; **(F–J)**. P2pal-18S facilitated gefitinib to increase expressions of E-cadherin and decrease expressions of vimentin; **(K)**. The heat map of gene expressions of vimentin, E-cadherin, Slug, Bcl-2 and Bax by different treatments compared with control group (value = 1) in PC-9 and PC-9-GR cells, respectively. *p < 0.05, **p < 0.01, ***p < 0.001.

It has been reported that the EMT phenotype and dysregulation of bypass signaling could initiate gefitinib resistance in NCSLC (Weng et al., 2019), which are also highly associated with PAR2 activation (Tsai et al., 2018). Therefore, we studied cell signaling pathway and EMT in P2pal-18S and gefitinib-treated cells for exploring the underlying mechanism of PAR2 inhibition in sensitizing NSCLC cells to gefitinib. With increasing resistance to gefitinib, there was a rising trend in phosphorylation of ERK (Figure 3A), suggesting the participation of ERK signaling in EGFR-TKI resistance. Furthermore, ERK signaling has been revealed to be modulated by the transactivation between PAR2 and EGFR in cancer cells (Darmoul et al., 2004; Shi et al., 2013). Our results further showed that P2pal-18S potentiated gefitinib inhibition in phosphorylation of ERK, suggesting that targeting PAR2 may inhibit EGFR transactivation and ERK activation to reverse gefitinib resistance in NSCLC (Figures 3B,C).

Alternatively, at mRNA and protein expression levels, E-cadherin was suppressed and vimentin was elevated in PC-9-GR cells, indicating the occurrence of EMT when gefitinib resistance were generated (Figures 3A,D). Meanwhile, we screened the gene expressions of EMT-related transcription factors, Slug, ZEB1, Snail and Twist, showing an enhancement in Slug and Twist expressions in PC-9-GR compared to PC-9 cells. Moreover, PC-9-GR cells possessed more spindle mesenchymal-like phenotypes whereas most of PC-9 cells were round epithelial-like cells, confirming EMT during the development of gefitinib resistance (Figure 3E). Importantly, our results uncovered that the cooperative effect of PAR2 and EGFR inhibitors in reversing EMT (Figures 3F–J). P2pal-18S markedly potentiated gefitinib to increase the protein expression of E-cadherin and decrease vimentin (∼2-fold) in both sensitive and resistant cells whereas gefitinib alone did not trigger such effects, suggesting PAR2 antagonism could assist gefitinib to inhibit EMT. The gene expression changes of vimentin, E-cadherin, Slug, Bcl-2 and Bax induced by P2pal-18S and gefitinib were also plotted and compared in the heat map, which revealed that the combination of PAR2 and EGFR blockade significantly prevented Slug-mediated EMT and apoptosis, compared to gefitinib alone (Figure 3K). Therefore, inhibition of PAR2 may suppress ERK activation and Slug-mediated EMT to overcome gefitinib resistance in NSCLC cells.

### Protease-Activated Receptor 2-Mediated β-Arrestin-Epidermal Growth Factor Receptor-ERK Signaling Axis Was Critical for Overcoming Gefitinib Resistance in Non-Small Cell Lung Cancer Cells

**FIGURE 4:**
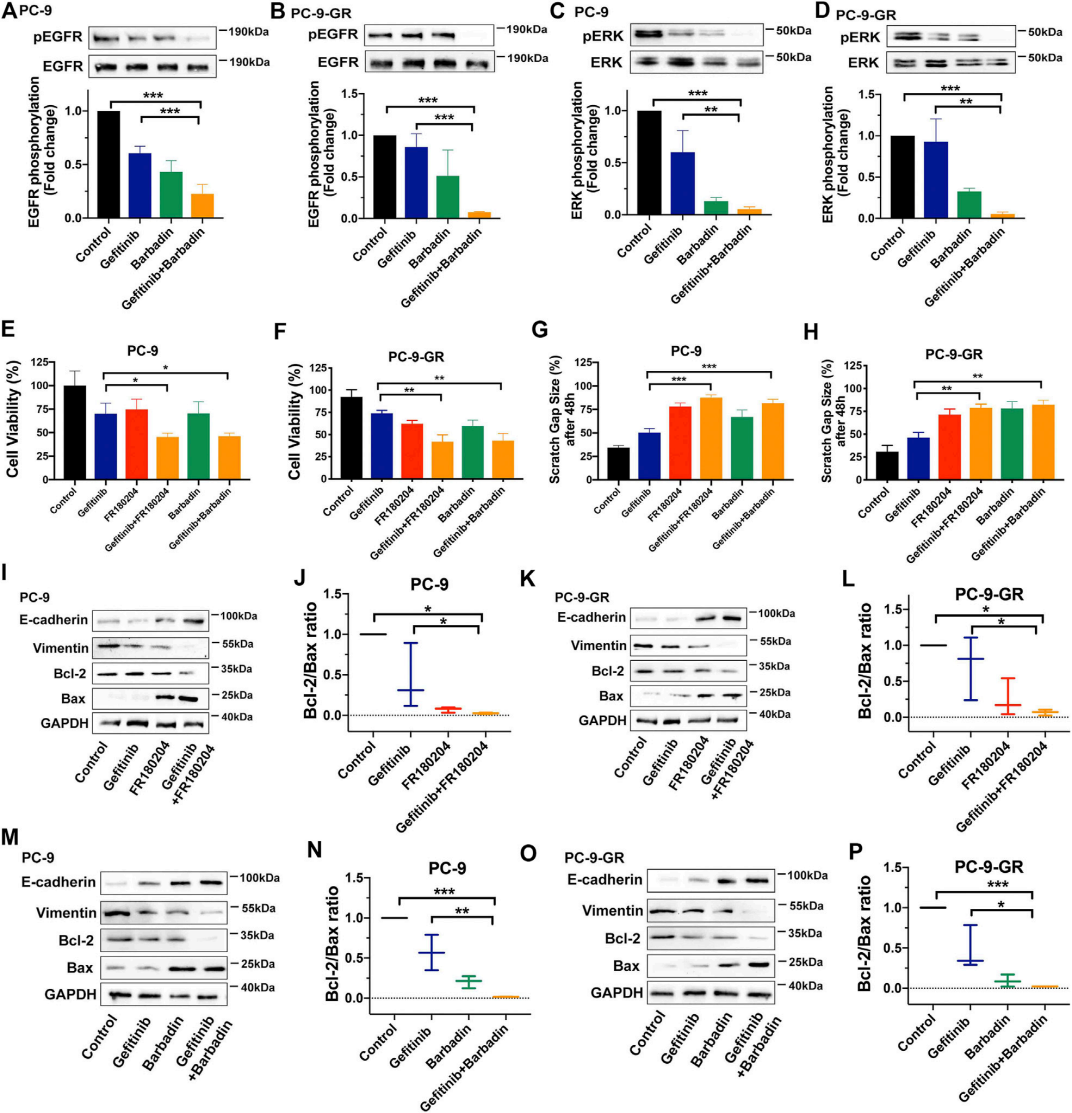
PAR2 reversed NSCLC cell resistant to gefitinib via β-arrestin-EGFR-ERK signaling. **(A–D)**. A β-arrestin inhibitor (Barbadin, 30 μM) facilitated gefitinib to block phosphorylation of EGFR and ERK in PC-9 **(A, C)** and PC-9-GR cells **(B, D)**; **(E–F)**. Either barbardin or ERK inhibitor (FR180204, 10 μM) largely enhanced inhibitory effects of gefitinib in cell viability of PC-9 **(E)** and PC-9-GR **(F)**; **(G–H)**. Barbadin or FR180204 also promoted gefitinib to attenuate cell migration of PC-9 **(G)** and PC-9-GR **(H)**; **(I–L)**. Addition of FR180204 assisted gefitinib to up-regulate or down-regulate expressions of E-cadheirn, vimentin, bcl-2 and bax, suggesting β-arrestin and ERK related to PAR2-mediated EMT and apoptosis **(I, K)**. The bcl-2/bax ratios were also calculated **(J, L)**; **(M–P)**. Barbadin also enhacanced gefitinib to modulate EMT- or apoptosis-related gene expressions in PC-9 **(M)** and PC-9-GR cells **(O)**, while bcl-2/bax ratios were presented **(M,P)**. *p < 0.05, **p < 0.01, ***p < 0.001.

For deeply understanding of its molecular mechanism, we looked into the role of the receptor transactivation-mediated signaling pathway in NSCLC cell functions related to gefitinib resistance. β-arrestin is considered as a crucial factor regulating EGFR transactivation while PAR2 could promote β-arrestin to drive EGFR transactivation and downstream ERK signaling (Köse, 2017). Similar to previous studies, barbadin as a β-arrestin1/2 inhibitor effectively facilitated gefitinib to impair phosphorylation of EGFR and ERK, uncovering the participation of β-arrestin in EGFR transactivation (Figures 4A–D). We then combined gefitinib with barbadin or an ERK inhibitor (FR180204) to treat PC-9 and PC-9-GR cells, in order to investigate participations of PAR2-mediated β-arrestin and ERK signaling in gefitinib resistance. As shown in Figures 4E–H and Supplementary Figure S4, either barbadin or FR180204 notably enhanced gefitinib inhibition in NSCLC cell viability and migration (∼2-fold) compared to gefitinib alone, indicating potential roles of β-arrestin and ERK signaling in reversing resistance to gefitinib. Furthermore, with inhibition of β-arrestin or ERK, EMT was suppressed by gefitinib treatments since there were increased expression of E-cadherin and decreased expression of vimentin (Figures 4I,K,M,O, Supplementary Figure S5). The bcl-2/bax ratio were also largely down-regulated when addition of barbadin or FR180204 into gefitinib in PC-9 and PC-9-GR cells, revealing participations of β-arrestin or ERK in reactivating gefitinib inhibition in apoptosis (Figures 4I–P). Thus, it suggested that PAR2 inhibition may reverse NSCLCs resistant to gefitinib through the β-arrestin-EGFR-ERK signaling.

### Protease-Activated Receptor 2 Inhibition Augmented Gefitinib to Attenuate Tumor Growth by Preventing ERK Activation *in vivo*

**FIGURE 5:**
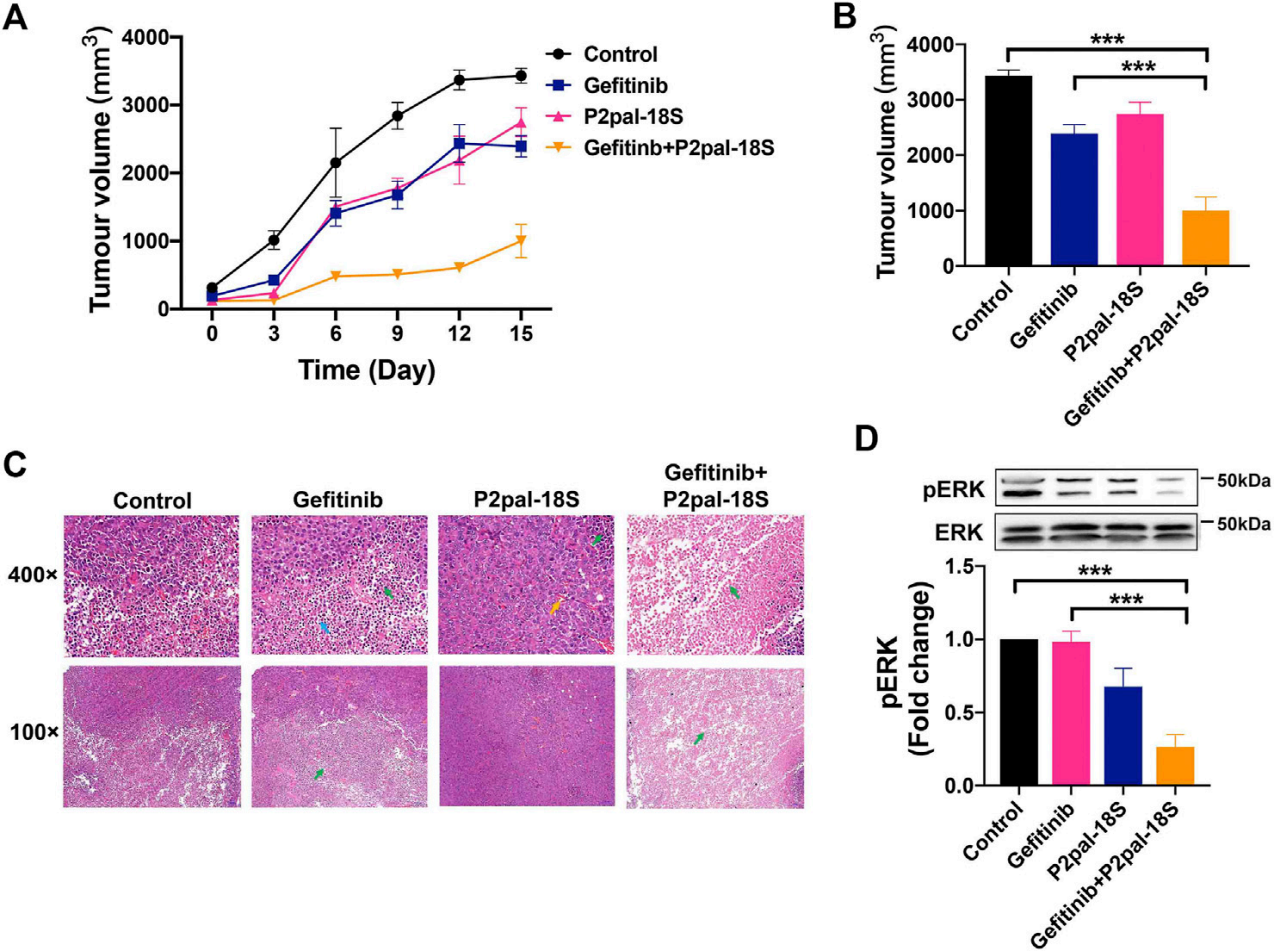
PAR2 inhibition reactivated gefitinib to inhibit tumor growth via ERK signaling *in vivo*. **(A)**. The combination of P2pal-18S and gefitinib significantly attenuated PC-9-GR tumor growth; **(B)**. P2pal-18S promoted gefitinib to decrease tumor growth whereas gefitinib exhibited little inhibitory effects; **(C)**. The H&E staining analysis of tumor tissues with different treatments were detected; **(D)**. P2pal-18S facilitated gefitinib to inhibit phosphorylation of ERK in PC-9-GR tumor tissues. Tumor cell necrosis (green), blood capillary (yellow), neutrophil (blue), ***p < 0.001.

The therapeutic benefit of PAR2 antagonism in reversing gefitinib resistance was further investigated in a PC-9-GR xenograft model. The combination treatment of P2pal-18S and gefitinib notably augmented the *in vivo* antitumour effect whereas gefitinib itself exhibited little therapeutic ability for 15 days (Figure 5A). P2pal-18S largely facilitated gefitinib to slow down and inhibit tumor growth with ∼3-fold decrease of tumor volume (Supplementary Figure S6, Figure 5B, ***p < 0.001). Moreover, H&E staining results showed that combination of P2pal-18S and gefitinib triggered abundant cell necrosis (∼90%, green arrow) in tumor tissues compared to negative control and gefitinib alone (Figure 5C). Cells in the tumor tissues from combination treatment group were loosely arranged with disintegration, lyzed nuclei and eosinophilic cell fragments, whereas there were only a small number of non-necrotic tumor cells.

To confirm the underlying mechanism of PAR2 inhibition *in vivo*, we studied the key regulatory factor, ERK, in tumor tissues from drug-treated xenograft mice. Unsurprisingly, the combination of P2pal-18S and gefitinib notably restricted protein levels of phospho-ERK (∼2.5 fold) in PC-9-GR tumors compared to gefitinib alone detected by western blot analysis (Figure 5D), suggesting that PAR2 inhibition may reactivate gefitinib to block PC-9-GR tumor growth via limiting ERK activation, which consistent with previous *in vitro* results.

## Discussion

GPCRs are the largest membrane receptor family and widely investigated drug targets, which exhibit a crosstalk with RTKs family including EGFR. Although GPCR-mediated EGFR transactivation was identified as a crucial mechanism in modulating a variety of tumor cell signaling and functions (Köse, 2017), therapeutic potentials of GPCRs have been rarely probed in EGFR-TKI resistance. A recent study showed that a GPCR membrane receptor, CXCR7, could facilitate NSCLC resistant to gefitinib via reactivating ERK signaling (Becker et al., 2019), but the possible participation of receptor transactivation in molecular mechanism has not been extensively reported. We found overexpression of PAR2 that belongs to GPCRs in lung tumor tissues from patients compared to normal controls while PAR2 expression was highly related to overall survival of patients, indicating a promising role of PAR2 in lung tumor progression (Figures 1A–C, Supplementary Figure S1). More importantly, we found that PAR2 expression was notably elevated in NSCLC cells during the generation of gefitinib resistance (Figure 1G) and this was consistent with a previous multiple analysis of GPCRs expressions in gefitinib-resistant NSCLC cells (Kuzumaki et al., 2012). PAR2 can transactivate EGFR (Kawao et al., 2005; Huang et al., 2013; Michel et al., 2014), and P2pal-18S as a PAR2 inhibitor cooperatively augmented gefitinib to attenuate phosphorylation of EGFR in both gefitinib-sensitive and -resistant NSCLC cells (Figures 1D,H), which suggested that the inhibition of receptor transactivation may affect drug resistance. Moreover, several studies have been reported that PAR2 could modulate numerous cancer cell functions, such as proliferation, migration and apoptosis, by EGFR transactivation or itself (Darmoul et al., 2004; Michel et al., 2014; Arakaki et al., 2018; Pawar et al., 2019). It is not hard to speculate that PAR2 inhibition may attenuate tumor cell signaling pathways and functions to reverse gefitinib resistance in NSCLC, but there is a lack of such investigations. Our findings showed that a synergic effect of the PAR2 inhibitor and gefitinib in preventing cell viability in gefitinib-sensitive cell PC-9, acquired resistant cell PC-9-GR and primary resistant cell A549 (Figures 1I–K), revealing targeting PAR2 could sensitive NSCLC responses to gefitinib. Moreover, PAR2 blockade exhibited a stronger sensitization effect in PC-9-GR cells than in PC-9 cells. This reversal effect of PAR2 inhibition was further uncovered in enhancing gefitinib to modulate NSCLC cell migration and apoptosis (Figure 2, Supplementary Figure S3). Moreover, a PAR2 inhibitor markedly reactivated gefitinib to prevent tumor growth in PC-9-GR xenograft mice (Figure 5), confirming that PAR2 inhibition could overcome gefitinib resistance *in vivo*.

Moreover, it has been reported that some cell signaling pathways and histological changes not only involved in transactivation of PAR2 and EGFR, but also promoted gefitinib resistance in NSCLC (Michel et al., 2014; Rotow and Bivona, 2017). However, rare studies attempted to linking signaling pathways in EGFR-TKI resistance to PAR2 regulation. ERK signaling as a compensatory downstream signaling promotes the development of gefitinib resistance whereas combination of MEK/ERK and EGFR inhibitors could prevent EGFR-TKI resistance (Tricker et al., 2015). Our results uncovered the involvement of ERK signaling upon EGFR transactivation in targeting PAR2 overcoming gefitinib, since phosphorylation of ERK was largely increased in gefitinib-resistant cells and PAR2 blockade promoted gefitinib to attenuate ERK activation (Figures 3A–C). Furthermore, we observed a loss of E-cadherin and an increase of vimentin, which are the hallmark of EMT, in gefitinib-resistant NSCLC cells compared to sensitive cells (Figures 3A,D). This is in accordance with previous reports on the existence of EMT as a histological transformation during generation of EGFR-TKI resistance (Weng et al., 2019). There are numerous attempts in exploring therapeutic approaches for targeting EMT to overcome EGFR-TKI resistance, e.g., metformin, gene knockout or pharmacological inhibition of FGFR or a transcription factor, Twist1 (Li et al., 2014; Raoof et al., 2019; Yochum et al., 2019). Meanwhile, EMT can be triggered by PAR2 activation in lung cancer cells and inhibition of PAR2 can stimulate prevention of EMT and cell migration (Sun et al., 2018; Tsai et al., 2018). When NSCLC became resistance to gefitinib, EMT could not be blocked by gefitinib whereas addition of a PAR2 inhibitor notably sensitized gefitinib to up-regulate E-cadherin and down-regulate vimentin, consequently overcoming EMT-related drug resistance (Figures 3F–K). Furthermore, a transcription factor Slug, but not ZEB1, Snail or Twist, actively involved in this PAR2-mediated EMT in gefitinib resistance (Figures 3D,K).

Alternatively, GPCRs have been discovered to stimulate key mediators, such as β-arrestin, SRC or MMP, to transactivate EGFR and downstream signaling (Bhola and Grandis, 2008; Köse, 2017). Our work also uncovered that β-arrestin mediated transactivation between PAR2 and EGFR in gefitinib-resistant NSCLC since inhibition of β-arrestin immediately impaired phosphorylation of EGFR and ERK (Figures 4A–D). Moreover, previous studies reported that a variety of PAR2-mediated cancer cell functions were depending on ERK signaling pathways (Yau et al., 2013; Michel et al., 2014; Jiang et al., 2017). Inhibition of either β-arrestin or ERK augmented gefitinib to impair EMT, migration and proliferation in PC-9-GR cells (Figure 4E–P), revealing β-arrestin-ERK signaling as a principal pathway for targeting PAR2 in reversing gefitinib-resistant NSCLC cells. The inhibition of PAR2 also notably facilitated gefitinib to slow down and block PC-9-GR tumor growth, while phosphorylation of ERK was markedly attenuated in tumor tissues from the combination treatment group, *in vivo* confirming targeting PAR2 could overcome gefitinib in NSCLC via ERK signaling pathway (Figure 5).

In summary, we first revealed that the novel therapeutic potential of PAR2 inhibition in reversing NSCLC resistance to gefitinib resistance and its molecular mechanism. PAR2 expression was markedly elevated in NSCLC tumor progression and gefitinib resistance, indicating the participation of PAR2 in NSCLC resistant to gefitinib. Targeting PAR2 facilitated the effect of gefitinib in EMT, cell proliferation, migration and apoptosis in gefitinib-sensitive and -resistant NSCLC cells through blocking β-arrestin-ERK signaling upon EGFR transactivation. The combination of a PAR2 inhibitor and gefitinib in PC-9-GR xenograft model exhibited larger inhibition of tumor growth and phosphorylation of ERK, confirming that the reversal effect of PAR2 inhibition as well as the underlying mechanism *in vivo*. This study not only suggested PAR2 as a novel therapeutical target for gefitinib resistance but also uncovered a new receptor-mediated mechanism for overcoming NSCLC resistant to gefitinib. Meanwhile, we provided a preclinical evidence for the combination of a PAR2 inhibitor and gefitinib as a promising approach to reverse drug resistance and improve the overall survival of NSCLC patients.

## Data Availability

The original contributions presented in the study are included in the article/Supplementary Material. Further inquiries can be directed to the corresponding authors.
