# Supplementary material for: Targeting PAR2 Overcomes Gefitinib Resistance in Non-Small-Cell Lung Cancer Cells Through Inhibition of EGFR Transactivation
Source: Front Pharmacol. 2021 Apr 22;12:625289. doi: 10.3389/fphar.2021.625289 (PMC8100583; doi:10.3389/fphar.2021.625289)
Supplement: Supplementary file 1 [file datasheet1.docx]

**Supporting materials: Targeting PAR2 overcomes gefitinib resistance in non-small-cell lung cancer cells through inhibition of** **EGFR transactivation**

**Figure S1**

Figure S1. GEO clinic database shows PAR2 expression in lung tumour tissues is higher than normal lung tissues, *** p < 0.001.

**Figure S2**

Figure S2. Cell viability detected after gefitinib treatment. (A). Human non-small lung cancer cells A549 shows primary resistance to gefitinib, IC_50_=19 μM; (B-C). Cell viability was measured after treated with gefitinib for 24 h, 48 h or 96 h in PC-9 (B) and PC-9-GR (C).

**Figure S3**

Figure S3. P2pal-18S facilitated gefitinib to promote cell apoptosis of PC-9 and PC-9-GR, detected by Annexin-FITC/PI staining assay.

**Figure S4**


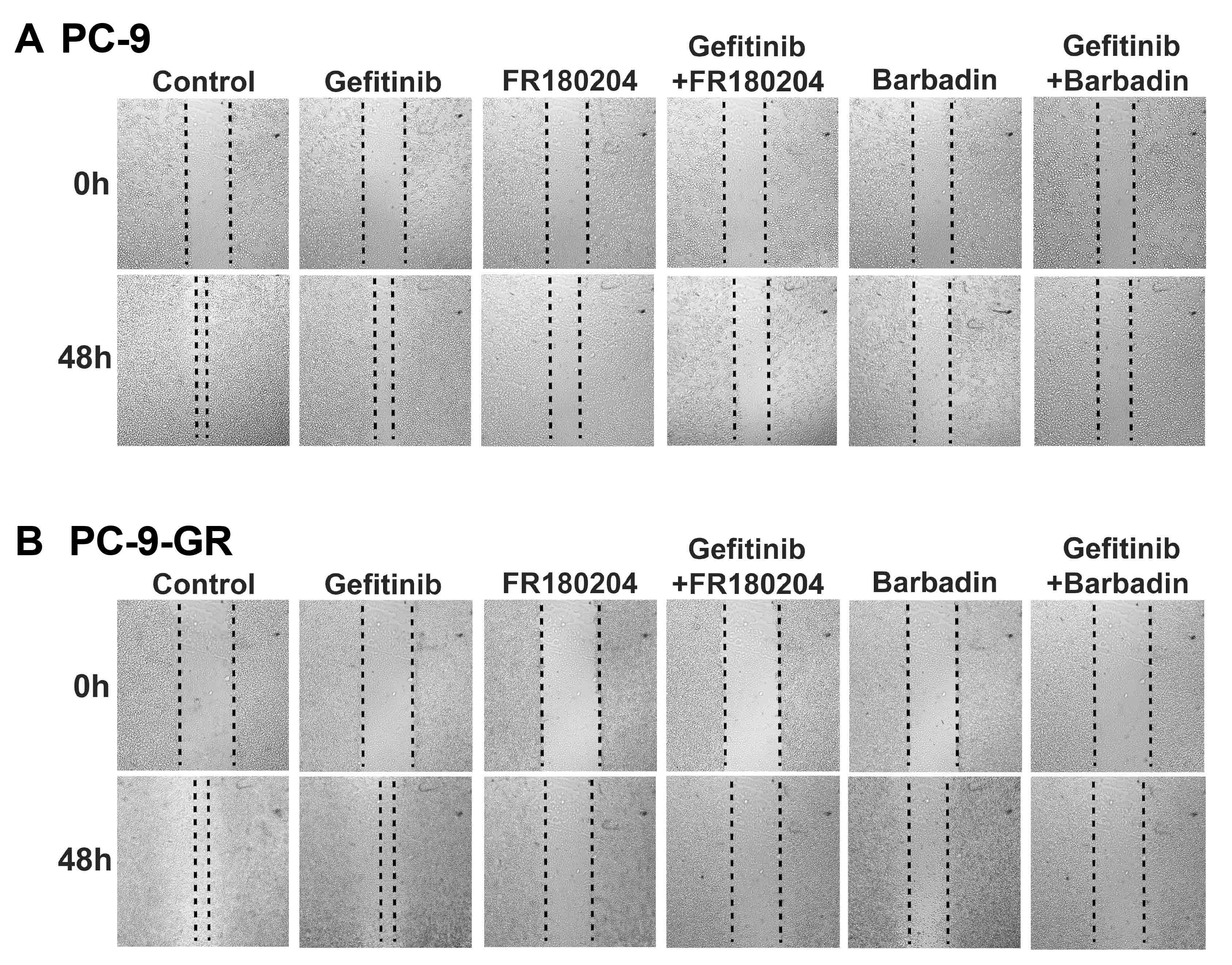


Figure S4. The β-arrestin inhibitor (Barbadin) or ERK inhibitor (FR180204) could promote gefitinib to block scratch gap closures in PC-9 (A) and PC-9-GR cells (B).

**Figure S5**

Figure S5. The quantitative analysis of E-cadherin and Vimentin expressions in combination of gefitinib and barbadin or FR180204 in PC-9 and PC-9-GR cells.

**Figure S6**


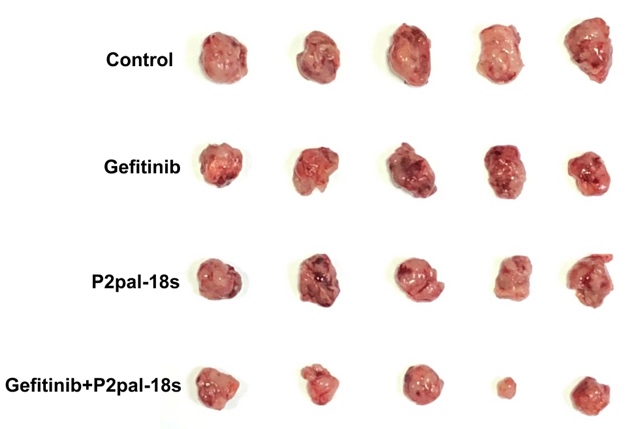


Figure S6. The comparison of tumour volume in different treatment groups.

**Table S1**

Primer sequences for real time-PCR.

| **Gene Symbol** | **Forward sequence** | **Reverse sequence** |
| --- | --- | --- |
| *ACTIN* | CATGTACGTTGCTATCCAGGC | CTCCTTAATGTCACGCACGAT |
| *F2RL1* | GGGTTTGCCAAGTAACGGC | GGGAACCAGATGACAGAGAGG |
| *E-CADHERIN* | TAACCGATCAGAATGAC | TTTGTCAGGGAGCTCAGGAT |
| *VIMENTIN* | GAGTCCACTGAGTACCGGAGAC | TGTAGGTGGCAATCTCAATGTC |
| *SLUG* | CTTTTTCTTGCCCTCACTGC | ACAGCAGCCAGATTCCTCAT |
| *ZEB1* | TGCACTGAGTGGAAAAGC | TGGTGATGCTGAAAGAGACG |
| *TWIST* | GGAGTCCGCAGTCTTACGAG | TCTGGAGGACCTGGTAGAGG |
| *SNAIL* | ACCCCACATCCTTCTCACTG | TACAAAAACCCACGCAGACA |
| *BAX* | TGGCAGCTGACATGTTTTCTGAC | TCACCCAACCACCCTGGTCTT |
| *BCL2* | TTTGAGTTCGGTGGGGTCAT | TGACTTCACTTGTGGCCCAG |
